# The association between *ST8SIA2* gene and behavioral phenotypes in children with autism spectrum disorder

**DOI:** 10.3389/fnbeh.2022.929878

**Published:** 2022-07-25

**Authors:** Xiaolei Yang, Lin Li, Xuejiao Chai, Jicheng Liu

**Affiliations:** ^1^Postdoctoral Workstation, Research Institute of Medical and Pharmacy, Qiqihar Medical University, Qiqihar, China; ^2^Department of Preventive Medicine, School of Public Health, Qiqihar Medical University, Qiqihar, China; ^3^Center for Prevention of Disease, Hospital of Traditional Chinese Medicine of Qiqihar, Qiqihar, China

**Keywords:** autism spectrum disorders, *ST8SIA2* gene, DNA methylation, behavioral phenotypes, pyrosequencing

## Abstract

**Objective:**

ST8 alpha-N-acetyl-neuraminide alpha-2,8-sialyltransferase 2 (*ST8SIA2*) encodes a type II membrane protein that is thought to catalyze the transfer of sialic acid (SA) from CMP-SA to N-linked oligosaccharides and glycoproteins. Some population and animal studies have indicated an association between the *ST8SIA2* gene and autism spectrum disorder (ASD). However, there is limited information on the correlation between *ST8SIA2* and autistic behavioral symptoms.

**Methods:**

In this study, 69 ASD and 76 normal control children who were age- and sex-matched were recruited. *ST8SIA2* expression and methylation levels were measured by reverse transcription quantitative real-time PCR and pyrosequencing, respectively, and the behavioral phenotypes of ASD children were assessed.

**Results:**

The ASD group had lower *ST8SIA2* gene expression levels than the control group [*t*_(0.05/2,143)_ = 2.582, *p* = 0.011]. Moreover, *ST8SIA2* expression levels were positively correlated with daily life skills (*r_*s*_* = 0.381, *p* = 0.008) and negatively associated with stereotyped behaviors in the ASD group (*r_*s*_* = -0.510, *p* = 0.004). The methylation levels of the Chr. 15: 92984625 and Chr. 15: 92998561 sites of the *ST8SIA2* gene in ASD children were higher than those of controls. The Chr. 15: 92984625 site was positively correlated with the stereotyped behaviors of ASD children (*r_*s*_* = 0.41, *p* = 0.039).

**Conclusion:**

This study provides a scientific basis to elucidate the relationship between the *ST8SIA2* gene and behavioral phenotypes of ASD.

## Introduction

Autism spectrum disorder (ASD) is an early onset developmental disorder characterized by deficits in communication and social interaction and restrictive or repetitive behaviors (Mitra et al., 2021). Although the etiology of ASD has not been fully elucidated, its occurrence is strongly associated with genetics and epigenetics (Ramaswami et al., 2020). Furthermore, patients with ASD have significant phenotypic and genetic heterogeneity, which poses a great challenge for etiological research and rehabilitation (Tordjman et al., 2018).

Glycosylation is an important modification of proteins and lipids that can regulate their function (Reily et al., 2019). More than 50% of proteins are post-translationally modified with glycans, and these modified proteins are involved in the occurrence of disease (Sato and Kitajima, 2021). Additionally, glycoproteins are key components of the neural extracellular matrix, so they participate in nearly every biological process in the developing brain (Dwyer and Esko, 2016). However, as a neurodevelopmental disease, the association of ASD with glycosylation is rarely reported. Clinical studies have found that patients with congenital glycosylation disorders exhibit ASD-like behavioral phenotypes. Thus, it will be of great significance to explore the relationship between the glycosylation process of the nervous system and ASD.

Sialic acid (SA) is an important monosaccharide unit of ganglioside and glycoprotein in the brain and an essential nutrient for brain development and cognition (Wang, 2009). SA often forms a polymer chain and adds to nerve cell adhesion molecule (NCAM) by glycosylation, which is involved in the development and plasticity of synapses and participates in the formation of neural networks. In the SA glycosylation process, polysialyltransferase [ST8 alpha-N-acetyl-neuraminide alpha-2,8-sialyltransferase 2 (ST8SIA2)] plays an important role. In our present research, we found that SA was decreased in ASD children and related to autistic behaviors (Yang et al., 2018). Moreover, the NCAM levels were low in ASD children. However, the reason for this finding has not been clarified. Therefore, we hypothesize that the *ST8SIA2* gene may play a significant role in resolving these questions. One study reported a patient with a behavioral disorder and ASD, who had a 520-kb chromosomal deletion at 15q26.1 encompassing the *ST8SIA2* gene (Kamien et al., 2014). In animal model research, *St8sia2* KO mice displayed decreased social motivational behavior, which is the core symptom of ASD (Calandreau et al., 2010). A single nucleotide polymorphism study showed the association between *St8sia2* and ASD (Hane et al., 2016). Nevertheless, the relationship between the *ST8SIA2* gene and ASD behavioral phenotypes has not been well illuminated. The present study was designed to better understand the expression of the *ST8SIA2* gene, and its methylation levels at the target loci, and the association with behavioral performance in ASD children. It will provide a novel view for comprehending symptoms of ASD.

## Material and methods

### Patients and evaluation of behavioral symptoms

We investigated 69 children with ASD and 76 age- and sex-matched typically developing children (age 2–6 years). All participants and controls were Han Chinese. The ASD children were diagnosed by two psychiatrists based on the criteria of the Diagnostic and Statistical Manual of Mental Disorders-Fifth Edition and the combined results of the Autism Diagnostic Interview–Revised (ADI-R) and Autism Diagnostic Observation Schedule. The exclusion criteria were children with genetic disorders, attention deficit hyperactivity disorder, tic disorders, and mental retardation. The control children were recruited from kindergartens, agreed to mental and neurological examinations, and did not exhibit any developmental or nervous system diseases. This study was approved by the Ethical Committee of Qiqihar Medical University (No. 201920) in 2019. In addition, an informed written consent form for participation in the study was signed by the parents or legal guardians of all study subjects.

The ASD children completed assessments of intellectual, social, and autistic behavior problems using the Peabody Picture Vocabulary Test (PPVT), the Autism Behavior Checklist (ABC), the Childhood Autism Rating Scale (CARS), the Vineland Adaptive Behavior Scale (VABS), the Social Responsiveness Scale (SRS), and the Infant-Junior Middle School Student’s Ability of Social Life Scale. The ABC contains five sub-scales (sensory, communication, language, social, and self-care skills), which are used to evaluate the severity of autistic symptoms and completed by the parents (Krug et al., 1980). The CARS is a commonly used tool for diagnosing ASD and its score of 30–36 is considered mild to moderate autism and 37–60 is severe autism (Meng et al., 2017). The PPVT scale examines children’s receptive vocabulary ability to evaluate the intellectual development of children (Weber et al., 2015). The SRS is a brief screening questionnaire for evaluating the severity of social skill deficits and describing other core features of ASD, which have been validated and shown to be reliable and to have good correspondence to the gold-standard ADI-R (Frye and Rossignol, 2016). The VABS is used for examining autistic core behaviors with a focus on communication and adaptive behaviors (Mandic-Maravic et al., 2015). The Infant-Junior Middle School Students Social-Life Abilities Scale is an adaptive behavioral scale, including self-help, locomotion, occupation, communication, socialization, and self-direction items. There are 132 items in this scale, and the child gets one point for each item for a total possible score of 132. The raw scores can be transformed into a standard score that is adjusted for age (Yuan et al., 2018). All behavioral assessment scales for ASD children were completed under professional face to face guidance.

### Measurement of *ST8SIA2* gene expression levels

Blood samples were obtained between 8:30 a.m. and 9:30 a.m. in the morning, and RNA and DNA were immediately extracted from fresh blood. Total RNA was extracted from fresh blood of the children with ASD and healthy controls using the RNAprep pure Blood Kit (TIANGEN BIOTECH (BEIJING) CO., LTD). A NanoDrop 2000 was used to test the concentration and purity of RNA. Subsequently, cDNA was reverse transcribed from RNA samples using the PrimeScript^®^ RT reagent Kit with gDNA Eraser according to the manufacturer’s instructions (TaKaRa Bio). The reverse transcription quantitative real-time PCR (RT–qPCR) was subsequently performed using the ABI-7500 System with the SYBR^®^ Select Master Mix (Applied Biosystems, Life-Technologies). The specific RT–qPCR primers for *ST8SIA2* were synthesized by Sangon Biotech (Shanghai) Co., Ltd. The primer sequences were as follows:

**Table:** 

*ST8SIA2*	forward:5′-TCCTGAAGCACCACGTCAAC-3
	reverse: 5′-TACATCAAGAGGCCGGTGGT-3′
*HS-ACTB*	forward: 5′-CCTGGCACCCAGCACAAT-3
	reverse: 5′-GGGCCGGACTCGTCATAC-3′

### The selection of *ST8SIA2* gene methylation sites

In our earlier research, a genome-wide DNA methylation analysis was performed in five pairs of ASD-discordant monozygotic twins. Subsequently, we mapped different DNA methylation sites of five pairs of ASD-discordant monozygotic twins in the gene network and obtained 4,057 differentially modified genes (Liang et al., 2019). Interestingly, *ST8SIA2* was present in the 4,057 intersecting genes. Then, we screened the target sites of the *ST8SIA2* gene using the difference method (calculated Δβ) and the fold-change method. The parameters were set to fold-change ≥ 2 or ≤0.5 and | Δβ| ≥ 0.1 as cutoffs. The target sites included Chr. 15: 92943919, Chr. 15: 92943938, Chr. 15: 92944418, Chr. 15: 92944439, Chr. 15: 92944460, Chr. 15: 92984625, Chr. 15: 92998561, Chr. 15: 92937874, and Chr. 15: 92957106. All of the above sites are located in the intronic region of the *ST8SIA2* gene.

### Methylation pyrosequencing

DNA was extracted from peripheral blood using QIAGEN kits (QIAampDNA Blood Mini Kit Q-51106). The DNA concentrations were determined using an ultramicro nucleic acid ultraviolet tester (Nanodrop 2000; Nanodrop Technologies; Thermo Fisher Scientific, Inc.). A sulfite reagent was used to modify the DNA, and the modified DNA was purified. Then, PCR was completed. The PCR conditions were set as follows: 95^°^C for 3 min; 40 cycles of 94^°^C for 30 s, 56^°^C for 30 s, and 72^°^C for 1 min; and 72^°^C for 7 min. To detect the *ST8SIA2* methylation levels, pyrophosphate sequencing analysis was performed using a Pyromark Q96 instrument. The PCR primers for methylation quantification are shown as follows:

**Table:** 

**Primer**	**Base sequence (5′ to 3′)**	**Biotin**
ST8SIA2-1F (150 bp)	ATGAGTTAGAGATAGTTGGGAGGATA	
ST8SIA2-1R	TATCTCACCCCACAATACCTCTCC	5′-biotin
ST8SIA2-1S	TGGTTATTTTTTGTTAGGG	
ST8SIA2-2F (111 bp)	GAAATAGTATGAAGAGGGGTGTGGA	
ST8SIA2-2R	CCCCTCAAACATTCCCAACAACA	5′-biotin
ST8SIA2-2S	AAGGTAGATGTATGGATT	
ST8SIA2-3F (130 bp)	GAGAAAAGGAGGGAAGTTTGTGATATTAAG	
ST8SIA2-3R	ATCCAATTTAAATATCTTTTCACTATCTAC	5′-biotin
ST8SIA2-3S	ATTAAGGTATAGGTGGTTT	

### Statistical analysis

All data were analyzed using SPSS 21.0 (SPSS Inc., Chicago, IL, United States). For the descriptive data, we calculated the means, medians, standard deviations, and interquartile ranges of demographic and outcome variables (independent *t*-test, paired *t*-test, and one-way ANOVA). The chi-square test was used to determine differences in the distribution of categorical variables in different groups. Methylation levels were analyzed using the paired *t*-test. The correlation among the *ST8SIA2* expression levels, methylation level, and behavioral phenotype of ASD children was determined by Pearson’s or Spearman’s correlation analysis. For all analyses, significance was set at a *p*-value of 0.05.

## Results

### Patient characteristics

We investigated 69 children with ASD (14 girls, 55 boys, age 4.47 ± 1.23 years) and 76 age- and sex-matched typically developing children (16 girls, 60 boys, age 4.59 ± 1.19 years). All participants and controls were Han Chinese. There was no significant difference in sex or age between the two groups [sex: *x*^2^ = 0.013, *p* = 0.91; age: *t*_(0.05/2,143)_ = 0.549, *p* = 0.584].

### *ST8SIA2* gene expression levels in the autism spectrum disorder and control groups

**FIGURE 1:**
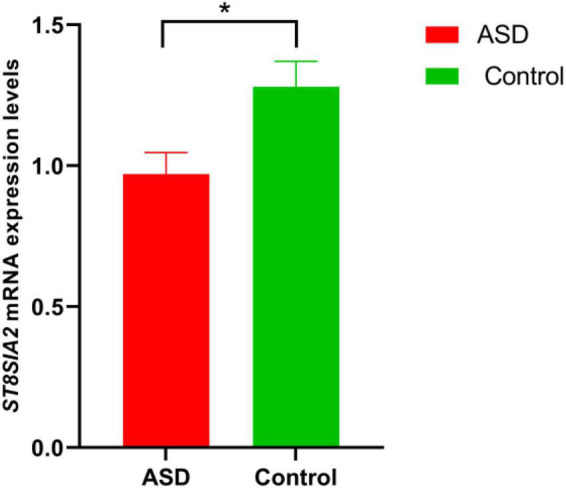
ST8 alpha-N-acetyl-neuraminide alpha-2,8-sialyltransferase 2 (*ST8SIA2)* mRNA levels in the autism spectrum disorder (ASD) and control groups (*N*_*ASD*_ = 69, *N*_*control*_ = 76). Data represented as means ± SEM. *p < 0.05 from the two dependent *t*-tests.

As shown in Figure 1, there were significant differences in *ST8SIA2* gene expression levels between the ASD and control groups. Moreover, the level of the *ST8SIA2* gene was lower in the ASD group than in the control group [ASD and control: 0.97 ± 0.64 and 1.28 ± 0.79, *t*_(0.05/2,143)_ = 2.582, *p* = 0.011] (Figure 1). Despite the diverse occurrence of ASD between sexes, there was no statistical difference in the expression of ST8SIA2 between the sexes by two-way ANOVA analysis [*F*_(1_,_142)_ = 1.625, *p* = 0.204]. Additionally, the expression of *ST8SIA2* was time-dependent, but there was no statistical difference among different ages through two-way ANOVA analysis [*F*_(4_,_135)_ = 1.259, *p* = 0.289].

### Relationship between *ST8SIA2* gene expression levels and autism spectrum disorder severity in children

As noted in Table 1, based on the scores of the CARS scale and levels of *ST8SIA2* in ASD children, the difference in *ST8SIA2* levels in different severities was statistically significant. Additionally, no significant difference in *ST8SIA2* levels was noted in children with different intelligence quotients of ASD.

**TABLE 1:** Relationship between the severity and intelligence development of autistic children and the ST8 alpha-N-acetyl-neuraminide alpha-2,8-sialyltransferase 2 (ST8SIA2) mRNA levels.

Item	*N*	*ST8SIA2* (Mean ± SD)	*F*	*p*
**ABC scores**				
≤53	19	1.21 ± 0.91		
54–66	21	0.79 ± 0.42	2.198	0.119
≥67	26	0.96 ± 0.53		
**CARS scores**				
<30	13	1.29 ± 0.77		
30–36	26	1.04 ± 0.73	4.166	**0.020**
37–60	29	0.73 ± 0.37		
**PPVT scores**				
<70	36	0.89 ± 0.63		
70–85	18	0.99 ± 0.82	0.424	0.656
>85	12	1.08 ± 0.41		

Three children did not complete the ABC scale, one child did not complete the CARS scale, and three children did not complete the PPVT scale.

The bold values represented statistical significance (p < 0.05).

### Relationship between *ST8SIA2* levels and behavior problems in autism spectrum disorder children

**FIGURE 2:**
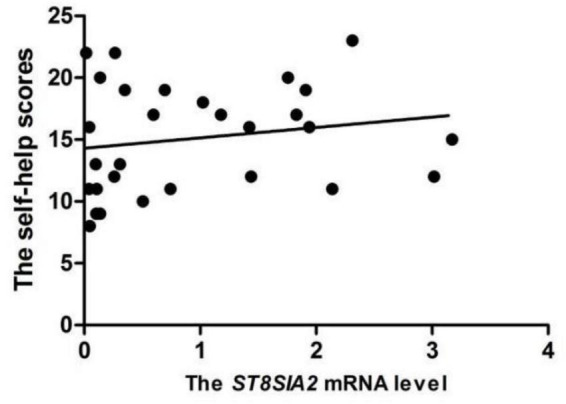
Correlation between *ST8SIA2* levels and self-help scores of Infant-Junior Middle School Students Social-Life Abilities Scale (*N*_*ASD*_ = 30, *r* = 0.462, *p* = 0.021 from Pearson’s correlation analysis).

There were 48 autistic children who finished VABS and SRS scales. A positive correlation was noted between the daily life skill score of the VABS scale and *ST8SIA2* levels in the evaluation of ASD children’s behavior problems (*r_*s*_* = 0.381, *p* = 0.008). This finding suggests that a higher expression level of the *ST8SIA2* gene corresponds to better daily life skills. However, there were no associations between the level of *ST8SIA2* and the SRS scores in the ASD group (Table 2). In the assessment of the CARS scale, the stereotype and sensory abnormality scores were negatively correlated with the *ST8SIA2* gene expression (*r_*s*_* = -0.510, *p* = 0.004). Thus, a lower level of the *ST8SIA2* expression indicates more serious stereotype behaviors in the ASD children. According to the results of the Infant-Junior Middle School Students Social-Life Abilities Scale in 30 ASD children, a positive correlation was noted between *ST8SIA*2 and the scores of self-help ability (*r* = 0.462, *p* = 0.021; as shown in Figure 2). These results indicate that a lower *ST8SIA2* expression level corresponds to worse self-help ability.

**TABLE 2:** Relationship between the *ST8SIA2* level and the adaptive behavior and social behavior in children with autism spectrum disorder (ASD).

Item	Scores (Mean ± SD)/*M* (*P*_25_–*P*_75_)	*r/r_*s*_*	*p*
**VABS total scores**	65 (58–72.75)	0.185	0.209
Communication	75.29 ± 23.08	0.103	0.486
Daily living skills	64 (61–76)	0.381	**0.008**
Socialization	58 (56–62.75)	0.174	0.238
Motor skills	91.05 ± 22.75	0.078	0.598
**SRS total scores**	89.02 ± 23.65	−0.033	0.861
Social awareness	10.80 ± 3.71	−0.053	0.781
Social cognition	18.83 ± 4.87	−0.266	0.155
Social communication	32.36 ± 8.11	0.035	0.856
Social motivation	14.85 ± 5.41	0.122	0.552
Autistic mannerisms	12.22 ± 6.69	−0.007	0.970

The bold values represented statistical significance (p < 0.05).

### Methylation levels of target sites in the *ST8SIA2* gene

We selected 30 ASD-control children pairs (age difference not exceeding 3 months in every pair and sex-matched, age from 2–6 years, 26 male pairs and 4 female pairs) which were from the population of ST8SIA2 mRNA expression test, and examined methylation levels at *ST8SIA2* gene sites. According to paired *t*-test result, the methylation levels of Chr. 15: 92984625 and Chr. 15:92998561 sites were significantly different between the ASD and control groups. The methylation levels in ASD children at these sites were significantly greater than those in control children [Chr. 15: 92984625: *t*_(0.05/2_,_29)_ = 3.81, *p* = 0.001; Chr. 15:92998561: *t*_(0.05/2_,_29)_ = 5.16, *p* < 0.001]. In addition, the methylation levels of Chr. 15: 92984630, Chr. 15: 92944439, and Chr. 15: 92944460 sites were higher than those in the control group, but the difference was not statistically significant (Table 3).

**TABLE 3:** Methylation level of ASD and controls at the detected site in *ST8SIA2* (mean ± SD).

Detected sit	ASD (%, *n* = 30)	Control (%, *n* = 30)	*t*	*P*
Chr. 15: 92984625	96.18 ± 048	95.41 ± 0.88	3.810	**0.001**
Chr. 15: 92984630	99.44 ± 1.04	99.11 ± 1.40	1.010	0.319
Chr. 15: 92984638	84.61 ± 4.30	84.77 ± 3.56	0.743	0.464
Chr. 15: 92944439	52.64 ± 3.06	52.55 ± 2.39	0.135	0.894
Chr. 15: 92944460	100	99.83 ± 0.69	1.343	0.190
Chr. 15: 92998561	99.51 ± 0.81	98.13 ± 1.29	5.160	**<0.001**

The bold values represented statistical significance (p < 0.05).

### Association between methylation levels at *ST8SIA2* gene sites and behavioral phenotypes in autism spectrum disorder children

Based on the above results, we analyzed the relationship between two significantly different methylation sites and behavioral problems in ASD children. It showed that no correlation between the Chr. 15:92998561 methylation level and the autism severity by comparing the case and control groups (*r* = -0.133, *p* = 0.484). There was no significant difference in the methylation levels of Chr. 15: 92998561 in ASD children with different levels of intellectual development [*F*_(2,27)_ = 1.211, *p* = 0.313]. In addition, no significant difference was found between the methylation levels of Chr. 15: 92984625 and the intellectual development levels of ASD children [*F*_(2,27)_ = 0.086, *p* = 0.917].

In addition, this study analyzed the relationship between the scores of communication skills, daily living skills, socialization, and motor skills in ASD children’s VABS scale and the methylation levels of Chr. 15: 92998561 and Chr. 15: 92984625. However, no statistically significant correlation was noted between them [Chr. 15: 92984625: *r*_(total scores)_ = -0.097, *p* = 0.611; *r*_(*communication skills*)_ = -0.021, *p* = 0.913; *r*_(*daily living skills*)_ = -0.258, *p* = 0.168; *r*_(*socialization*)_ = 0.044, *p* = 0.818; *r*_(*motor skills*)_ = 0.023, *p* = 0.904; Chr. 15: 92998561: *r*_(total scores)_ = 0.059, *p* = 0.758; *r*_(*communication skills*)_ = 0.011, *p* = 0.954; *r*_(*daily living skills*)_ = 0.102, *p* = 0.591; *r*_(*socialization*)_ = 0.057, *p* = 0.765; *r*_(*motor skills*)_ = 0.111, *p* = 0.56]. In addition, the methylation levels of Chr. 15: 92998561 and Chr. 15: 92984625 were not associated with the scores of the SRS [*r*_(*Chr.* 15: 92998561)_ = -0.105, *p* = 0.586; *r*_(*Chr.* 15: 92984625)_ = 0.019, *p* = 0.921].

In the Spearman correlation analysis, the results indicate that the methylation levels at the Chr. 15: 92984625 site were positively correlated with the stereotyped behavior scores of the ADI-R scale (*r_*s*_* = 0.41, *p* = 0.039). Thus, a greater methylation level of this site corresponds to a more serious stereotyped behavior. Furthermore, the methylation levels of the Chr. 15: 92984630 site were positively correlated with the scores of independent living ability and operational ability in the Infant-Junior Middle School Student’s Ability of Social Life Scale (*r_*s*_* = 0.478, *p* = 0.014; *r_*s*_* = 0.424, *p* = 0.031).

## Discussion

In this study, we explored *ST8SIA2* gene expression and methylation levels between the ASD and control groups and analyzed the relationship between *ST8SIA2* and the behavioral phenotypes of ASD children. *ST8SIA2* gene expression levels in the ASD group were lower than those in the control group. Moreover, these levels were associated with the severity, daily life skills, stereotype abnormality, sensory abnormalities, and self-help ability of ASD children. The ASD children had greater methylation levels of Chr. 15: 92984625 and Chr. 15: 92998561 sites than controls. Furthermore, Chr. 15: 92984625 was positively correlated with the stereotyped behavior scores on the ADI-R scale.

The *ST8SIA2* gene is involved in the synthesis of polysialyltransferase (ST8SIAII) and plays an important role in the glycosylation of NCAM. Some studies have reported that NCAM is correlated with ASD and autistic children’s behaviors (Shaw et al., 2014; Zhang et al., 2014; Yang et al., 2019). Moreover, genome-wide studies among normal individuals and patients with mental disorders reveal that *ST8SIA2* may be a candidate gene for ASD (Anney et al., 2010). In an intronic single nucleotide polymorphism (iSNP) study of the *ST8SIA2* gene, *ST8SIA2* was found to be related to ASD (Hane et al., 2016). Although some animal and patient studies reported that the *ST8SIA2* gene was associated with ASD, there are few reports on *ST8SIA2* mRNA levels in patients with ASD. In the current study, we found that the *ST8SIA2* expression of ASD children was decreased in comparison with that of control children. In animal research, it reported that ST8SIA2 expressed time-dependent, which is mainly expressed in embryonic and early postnatal mice. Hence, we detected whether there were differences in its expression between different age groups. Unfortunately, no statistical difference was discovered. Autism shows a striking male bias in prevalence, with approximately 4 affected men for every 1 affected female (Werling and Geschwind, 2013). It is noteworthy that developmental neurogenetics and multimodal neuroimaging manifested sex difference in ASD (Chen and Van Horn, 2017). Recent findings reveal that women with ASD exhibit more intellectual and behavioral problems compared with their male counterparts. Based on the above, we examined the expression of *ST8SIA2* in different sexes, but no expected results were obtained. This might be related to the small number of female sample participating in this study.

In addition, we explored the correlation between this gene and the behavioral problems of ASD children. This information will provide more scientific evidence to elucidate the relationship between the *ST8SIA2* gene and ASD. The *ST8SIA2* gene levels were low in the ASD groups and associated with the symptom severity in ASD children. According to our results, the expression levels of this gene may be lower in ASD children with more serious symptoms. In animal studies, the knockout of *St8sia2* was found to cause social problems in mice. However, in the behavioral evaluation of ASD children, we did not find a correlation between social behaviors and *ST8SIA2* gene expression. Interestingly, ASD children with low *ST8SIA2* gene expression levels exhibited serious stereotyped behaviors. Stereotyped behaviors are the core symptom of ASD and have been a hot spot in the study of the behavioral phenotype of autistic children, which has seriously affected children’s lives and studies and even made some comorbidities to occur (Kang et al., 2017; Bhandari et al., 2020). In addition, a positive correlation was noted between the *ST8SIA*2 expression and the self-help abilities and daily life skills of ASD children. The self-help ability is a serious problem among children with ASD, which causes difficulties in their school life and social activities (Bal et al., 2015; Chi and Lin, 2021). Based on these findings, the *ST8SIA2* gene has clinical value in elucidating the behavioral problems of patients with ASD, especially the self-care ability and stereotype behavior.

To explain the low *ST8SIA2* gene expression levels in ASD children, we tested the methylation levels at *ST8SIA2* gene sites. These sites were obtained from a genome-wide DNA methylation analysis, which was performed using five pairs of ASD-discordant monozygotic twins. These samples are high-quality samples for disease genetics and phenotype research, especially for non-cooccurrence cases of identical twins, which have identical genetic material and non-cooccurrence phenotype (Castillo-Fernandez et al., 2014). These samples are ideal for disease epigenetics research (Pallister et al., 2014). Therefore, the *ST8SIA2* gene sites from the MZ sample were tested and verified in sporadic ASD cases and provided a scientific basis to explore the correlation between the *ST8SIA2* gene and ASD. The 30 pairs of ASD and control children whose methylation levels were assessed in this study were age- and sex-matched, and the age difference of each pair was not greater than 3 months. We found that the methylation levels of Chr. 15: 92984625 and Chr. 15: 92998561 sites were statistically significant in the ASD sporadic cases. Interestingly, the methylation levels of the Chr. 15:92984625 site were positively correlated with the stereotyped behavior of ASD children, which is consistent with the relationship between the *ST8SIA2* gene expression and behavioral phenotypes. Furthermore, the methylation level of the Chr. 15: 92984630 site was positively correlated with the independent living ability, which is similar to the correlation between self-help ability and *ST8SIA2* gene expression. Although these sites are located in the intron region, they are valuable in explaining the association of the *ST8SIA2* gene with ASD behavioral phenotypes.

To some extent, autism does not affect life span, so it is difficult to obtain brain tissue samples in the etiology research of ASD. Based on the presence of the blood-brain barrier, some neural factors and biomolecules in the peripheral blood can indirectly reflect the expression levels in the brain tissue (Qin et al., 2016; Eshraghi and Davies, 2020; Kealy et al., 2020). The extraction of RNA and DNA from the peripheral blood is a less harmful way to ASD and control children. Furthermore, there were numerous studies investigating the association between behavioral performance and biomolecular levels in the peripheral blood of patients with ASD (Patrick and Ames, 2014; Hewitson and Mathews, 2021). Blood-based gene expression could divine infants and toddlers with autism, meanwhile, the alteration of gene expression in the peripheral blood of ASD is related to the core symptoms of autism (such as social impairment and stereotypical behaviors; Makinodan et al., 2017; Norouzi Ofogh and Rasoolijazi, 2021). Therefore, this study provides evidence for the etiological study of ASD. This study had some limitations. First, due to the small number of female participants, we could not explain the expression differences in the *ST8SIA2* gene in the sexes of the ASD and control groups. Second, this study used peripheral blood samples of ASD and control children; therefore, it was limited to demonstrating the relevance of *ST8SIA2* and ASD due to the lack of brain tissue samples. Third, we detected only a few intronic sites in the *ST8SIA2* gene. To clarify the role of the *ST8SIA2* gene in ASD more clearly, methylation levels of CpG island-*ST8SIA2* gene promoters should also be assessed.

## Data availability statement

The original contributions presented in this study are included in the article/supplementary material, further inquiries can be directed to the corresponding author.

## Ethics statement

The studies involving human participants were reviewed and approved by Ethics Committee of Qiqihar Medical University. Written informed consent to participate in this study was provided by the participants or their legal guardian/next of kin.

## Author contributions

XY: data analysis, evaluation of behavioral symptoms, and writing original draft. LL: measurement of *ST8SIA2* gene mRNA expression and methylation. XC: project administration. JL: review and editing the manuscript. All authors contributed to the article and approved the submitted version.

## Conflict of interest

The authors declare that the research was conducted in the absence of any commercial or financial relationships that could be construed as a potential conflict of interest.

## Publisher’s note

All claims expressed in this article are solely those of the authors and do not necessarily represent those of their affiliated organizations, or those of the publisher, the editors and the reviewers. Any product that may be evaluated in this article, or claim that may be made by its manufacturer, is not guaranteed or endorsed by the publisher.
